# Geographical and socio-economic inequalities in years of life lost across Norwegian municipalities and city districts in 2019: an ecological registry-based study

**DOI:** 10.1093/eurpub/ckaf086

**Published:** 2025-09-18

**Authors:** Hege Breivik, Ingeborg Forthun, Ann K S Knudsen, Lode van der Velde, Carl M Baravelli

**Affiliations:** Institute of Health and Society, University of Oslo, Oslo, Norway; Department of Disease Burden, Norwegian Institute of Public Health, Bergen, Norway; Department of Disease Burden, Norwegian Institute of Public Health, Bergen, Norway; Department of Global Public Health, Karolinska Institutet, Stockholm, Sweden; Department of Disease Burden, Norwegian Institute of Public Health, Bergen, Norway

## Abstract

Understanding local level impact of socio-economic and spatial disparities on health outcomes is crucial for informing effective public health interventions. This study examines the association between socio-economic factors, centrality, and premature mortality—measured as years of life lost (YLLs)—across Norwegian municipalities. We conducted an ecological, cross-sectional registry-based study across municipalities and districts, each with populations exceeding 1000 as of 1 January 2019. Data on mortality, demographics, education, income, and centrality were sourced from Statistics Norway. All-cause YLLs were calculated by multiplying age-specific mortality numbers by aspirational life expectancy from the Global Burden of Disease 2019 life tables. Municipalities were divided into quartiles based on a composite socio-economic position (SEP) score that integrated education and income, and grouped into centrality categories. Mixed-effects negative binomial regression models, crude and adjusted for age categories and sex, evaluated both relative and absolute associations. The lowest SEP quartile, assessed with a composite SEP score, had a 15% higher YLL rate compared to the highest quartile [incidence rate ratio (IRR) = 1.15; 95% confidence interval (CI) = 1.07–1.24], amounting to an absolute difference of 2127 YLLs per 100 000 population. Similarly, the least central quartile exhibited a 15% higher YLL rate compared to the most central one (IRR = 1.15; 95% CI = 1.09–1.21), translating to an absolute difference of 2057 YLLs per 100 000 population. There are substantial inequalities in premature mortality across Norwegian municipalities, strongly linked to municipal SEP and centrality.

## Introduction

Norway has long-standing policies to reduce social health inequalities [1] and regional policies to ensure equal access to healthcare, education, and employment across regions [2]. Despite this, significant disparities in health outcomes persist [3–5]. Although public health in Norway has improved, geographical and socio-economic inequalities remain pronounced [3, 4, 6]. These spatial and social disparities often align, influenced by regional economic structures, education systems, income levels, and socio-cultural conditions [7]. Additionally, a clear socio-economic gradient exists where lower education, income, and occupational status are linked with poorer living conditions, psychosocial stress, and higher morbidity and mortality [8, 9].

The 2012 Public Health Act [1] aims to disrupt the association between socio-economic factors and health outcomes by requiring municipalities to actively promote health, prevent disease, and address environmental health issues. This includes managing air and water quality, noise levels, hazardous substance exposures, and overall living conditions. Additionally, the Act emphasizes ensuring health equity and mandates regular monitoring of local health statuses [1]. This necessitates local health authorities to know and address specific community health needs. Local research can reveal health inequalities often missed in national studies, supporting local health authorities [3]. The Global Burden of Disease (GBD) Study [10] provides a framework for assessing inequalities, highlighting years of life lost (YLLs) as a key metric [10, 11]. While GBD offers county-level YLL estimates in Norway, more granular municipal estimates would enhance their utility for local public health initiatives. This study addresses this gap by (i) investigating YLL inequalities across Norwegian municipalities, including city districts in the four largest cities; (ii) exploring associations between socio-economic factors and YLLs; and (iii) evaluating urban-rural differences in these associations.

## Methods

### Study design and data sources

This ecological study utilized registry-based data for all registered residents in Norwegian municipalities and districts as of 1 January 2019. At that time, Norway was divided into four health regions, 18 counties, and 422 municipalities, which included 40 districts within the four largest cities. We used individual-level data from Statistics Norway, gathered from the Population Register, the National Education Database, and the Income Register. This data covered demographics, all-cause mortality, socio-economic indicators (education, post-tax income, and household size), and publicly available area-level centrality [12]. To ensure privacy and reduce bias, we included only areas with populations of 1000 or more, resulting in a total of 425 units analysed: 388 municipalities and 37 city districts (18 in Oslo, eight in Bergen, seven in Stavanger/Sandnes, and four in Trondheim). These city districts represent 25% of the national population, thereby enhancing representativeness. Throughout the study, all units are collectively referred to as ‘municipalities.

### Outcome

The outcome was all-cause premature mortality, measured as YLL rates per 100 000 population. This metric captures the burden of disease by measuring years lost due to premature death [11]. YLL was chosen over age-standardized mortality rates because it considers both the frequency and age of death, assigning greater weight to deaths occurring at younger ages, making it particularly useful for identifying vulnerable and disadvantaged groups at increased risk of early death [11, 13]. Municipality-specific YLL rates were calculated in four steps [13]: (i) age-specific death and population counts in five-year age groups (<1, 1–4, 5–9 … 85+); (ii) crude YLLs by multiplying age-specific deaths by corresponding residual life expectancy from the GBD 2019 life tables [14]; (iii) municipality-specific YLL rates per 100 000 by summing crude YLLs and population counts across age groups and dividing by the total population; (iv) age-standardized YLL rate per 100 000 population by multiplying age-specific YLL rates by the 2019 Norwegian standard population and summing them across age groups, using the formula: age-standardized YLL rate = Σ age-specific YLL rate × age-specific weight [14].

### Socio-economic variables

This study used socio-economic position (SEP) to reflect the broader socio-economic context of municipalities, assessed via linked individual data on education and income. Educational attainment was categorized using the International Standard Classification of Education (ISCED) levels 0–2 [15], identifying those aged 20+ with only primary or lower secondary education, a compulsory 10-year period as of 2019. Income was measured as the median equivalized household income post-tax per municipality (as of 31 December 2018) [16, 17], adjusted for household composition and accounting for disposable income [18].

To capture the combined contribution of education and income, which are highly correlated, and provide a single, easily interpretable metric, we developed a composite SEP score based on the Handbook on Constructing Composite Indicators [19] (detailed in the Supplementary Material, page 1). This composite SEP score offers a more integrated and comprehensive measure of SEP than separate measures, more effectively capturing its multifaceted nature [19, 20]. Sensitivity analysis of different weighting structures between education and income were conducted and showed no significant impact on the findings (see Supplementary Table S2). The composite score was divided into quartiles, with Q1 representing ‘high SEP’ and Q4 representing ‘low SEP’, corresponding to higher and lower education and income levels, respectively. We also conducted separate analyses for education and income, which are provided in Supplementary Table S1.

### Centrality groups

Using Statistics Norway’s 2020 centrality classification, municipalities were grouped into three centrality categories: urban (classes 1–2), intermediate (classes 3–4), and rural (classes 5–6) [12]. These are referenced as Group 1 for urban, Group 2 for intermediate, and Group 3 for rural in the tables. This classification is based on a centrality index considering travel time to workplaces and services from around 13 500 inhabited basic districts, aggregated and weighted by residents at the municipal level [21].

### Additional covariates

Female-to-male ratio and the proportions of residents in age categories 0–24, 25–39, 40–59, 60–79, and 80+ years per municipality were additionally assessed.

### Statistical analysis

Baseline municipal characteristics were described by SEP score quartiles. For normally distributed variables (e.g. age, sex), means and standard deviations were reported; medians and interquartile ranges were used for skewed distributions (e.g. population size); and frequencies and percentages were presented for categorical variables (e.g. municipalities and districts by SEP quartiles and centrality groups).

Associations between socio-economic factors, centrality, and YLLs were evaluated through both relative and absolute inequalities. We employed mixed-effects negative binomial regression with robust standard errors, using two models: Model 1 (crude) and Model 2 (adjusted for age categories and sex, serving as the main model), see Supplementary Material, page 2 for specifications. Incidence rate ratios (IRRs) for YLLs, with 95% confidence intervals (CIs), were reported using the high SEP quartile (Q1) as the reference and the most central group (group 1) as the centrality reference. Health regions (South-East, West, Mid, North) were included as a random intercept to account for the hierarchical structure of municipalities within health regions [22], capturing baseline YLL variations and unobserved heterogeneity related to educational attainment and income. Absolute inequalities were assessed by examining differences in mean YLLs across SEP quartiles and centrality groups, both crude and adjusted for sex and age categories using Model 2. Post-estimation margins for Poisson distributions were reported with 95% CIs. For a detailed description of model fit, please see Supplementary Material, page 1.

To assess potential bias from small population sizes, sensitivity analyses excluded municipalities and districts with fewer than 2000, 4000, and 10 000 residents, detailed in Supplementary Material, page 2.

To assess the impact of socio-economic disparities on premature mortality, we calculated the Population Attributable Fraction (PAF) [23] to estimate the proportion of YLLs attributable to these disparities at the municipal level. We used the composite SEP score, combining educational attainment and income, to enhance the PAF’s sensitivity and interpretive value. This calculation illustrates the potential reduction in the YLL burden if disparities were equalized to the highest SEP level. Additional calculation details, including statistical models and CI estimation, are available in the Supplementary Material, page 2.

We used a bubble plot (two-way scatterplot) to visualize the relationship between SEP (continuous) and age-standardized YLL rates per 100 000 population with marker size proportional to the square root of the population and colours indicating health regions. A linear fit line with 95% CIs was added to illustrate the trend. The plot was generated using Stata’s <twoway> function.

To further visualize the geographical distribution of SEP, age-standardized YLLs, and the relationship between the two, univariate, and bivariate choropleth maps were created using QGIS 3.36.2 Maidenhead.

### Software

Stata/MP version 18 for Windows was used for all statistical analysis.

### Ethical approval

Ethical approval was obtained from the Norwegian Regional Committees for Medical and Health Research Ethics (reference number: 2013/2394).

## Results

Table 1 presents descriptive statistics by composite SEP score quartiles at the municipal and district level. Municipalities were evenly distributed across quartiles, while 67.6% of city districts fell in the high SEP quartile (Q1). Gender distribution was consistent across quartiles, while older age groups were more prevalent in lower SEP quartiles, with 23.8% aged 60–79 and 6.2% aged 80+ in Q4, versus 18.7% and 4.5% in Q1. Median population size decreased from 15 877 in Q1 to 2909 in Q4. Urban areas were largely in Q1 (65.1%), while rural areas were more common in Q4 (34.4%). Regionally, the South-East spanned all quartiles but was notable in Q3 and Q4, the West in Q1 and Q2, Mid Norway in Q2 and Q3, and the North in low SEP (Q4).

**Table 1.** Descriptive statistics by composite SEP score quartiles at the municipal and district level

	SEP Q1 (high SEP)	SEP Q2	SEP Q3	SEP Q4 (low SEP)
Municipalities, *n* (%)	82 (21.1)	100 (25.8)	106 (27.3)	100 (25.8)
Districts, *n* (%)	25 (67.6)	6 (16.2)	0	6 (16.2)
Education ISCED 0–2, mean %	17.5	20.9	23.7	28.6
Income, mean (NOK)	458 204	421 591	407 912	386 558
Sex, mean %				
Males	50.6	51.0	50.8	51.6
Females	49.4	49.0	49.2	48.4
Age groups, mean %	–	–	–	–
0–24	31.0	30.0	28.8	27.0
25–39	19.6	17.6	16.7	16.4
40–59	26.3	25.9	26.4	26.7
60–79	18.7	21.2	22.6	23.8
80+	4.5	5.4	5.5	6.2
Population, median [interquartile range (IQR)]	15 877 (4905–29 254)	5716 (2445–12 062)	5771 (2988–12 187)	2909 (1530–6155)
Centrality, no. of municipalities/districts (%)	–	–	–	–
Group 1 (urban)	43 (65.1)	10 (15.1)	5 (7.6)	8 (12.1)
Group 2 (intermediate)	38 (22.8)	47 (28.1)	50 (30.0)	32 (19.2)
Group 3 (rural)	26 (13.5)	49 (25.5)	51 (26.6)	66 (34.4)
Country of origin, mean %				
Norwegian born	87.2	88.9	89.6	88.0
Foreign born	12.8	11.1	10.4	12.0
Health regions, no. of municipalities/districts (%)	–	–	–	–
South-East	37 (24.2)	28 (18.3)	41 (26.8)	47 (30.7)
West	58 (38.4)	55 (36.4)	32 (21.2)	6 (3.8)
Mid	8 (18.2)	17 (38.6)	13 (29.5)	6 (13.6)
North	4 (5.2)	6 (7.8)	20 (26.0)	47 (61.0)

Notes: Municipalities and districts follow the 2019 classification, with districts being city areas in the four largest cities. SEP is evaluated via a composite score of educational attainment and income, where high SEP quartiles feature the highest levels, and low SEP quartiles the lowest. Educational attainment reflects the percentage with only lower secondary education or less (ISCED 0–2) per area. Income is measured by the median equivalized household income after tax. ‘Mean %’ indicates the average percentage of individuals in each category (education, income, sex, age groups, country of origin) across SEP quartiles. Centrality categories (urban–intermediate–rural) are derived from Statistics Norway’s classifications. NOK, Norwegian kroner.

**Figure 1.**
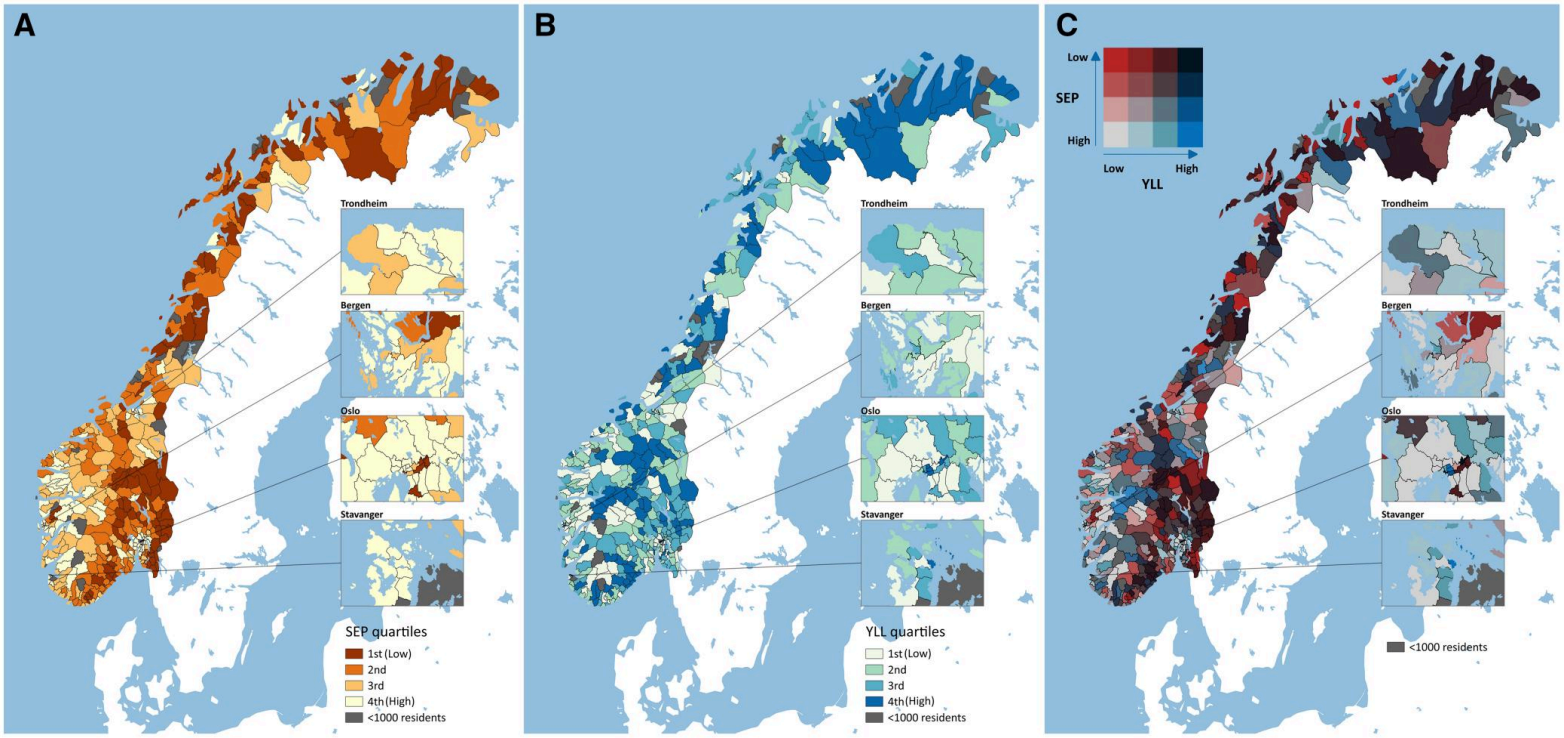
Geographical distribution of municipalities and districts by SEP quartiles (A), age-standardized YLL quartiles (B), and bivariate relationship between SEP and YLL (C). Notes: Panel A shows the geographical distribution of municipalities and districts divided into SEP quartiles, while Panel B displays age-standardized YLL rates per 100 000 population, organized into quartiles. Panel C presents a bivariate map that combines municipal SEP and YLL quartiles to illustrate their spatial relationship, highlighting areas where high or low SEP aligns with high or low YLLs.

Figure 1 depicts the geographical distribution and variations of municipalities by SEP quartiles (panel A), age-standardized YLL quartiles (panel B), and the cross-tabulation between municipal SEP and age-standardized YLL quartiles (panel C).

**Figure 2.**
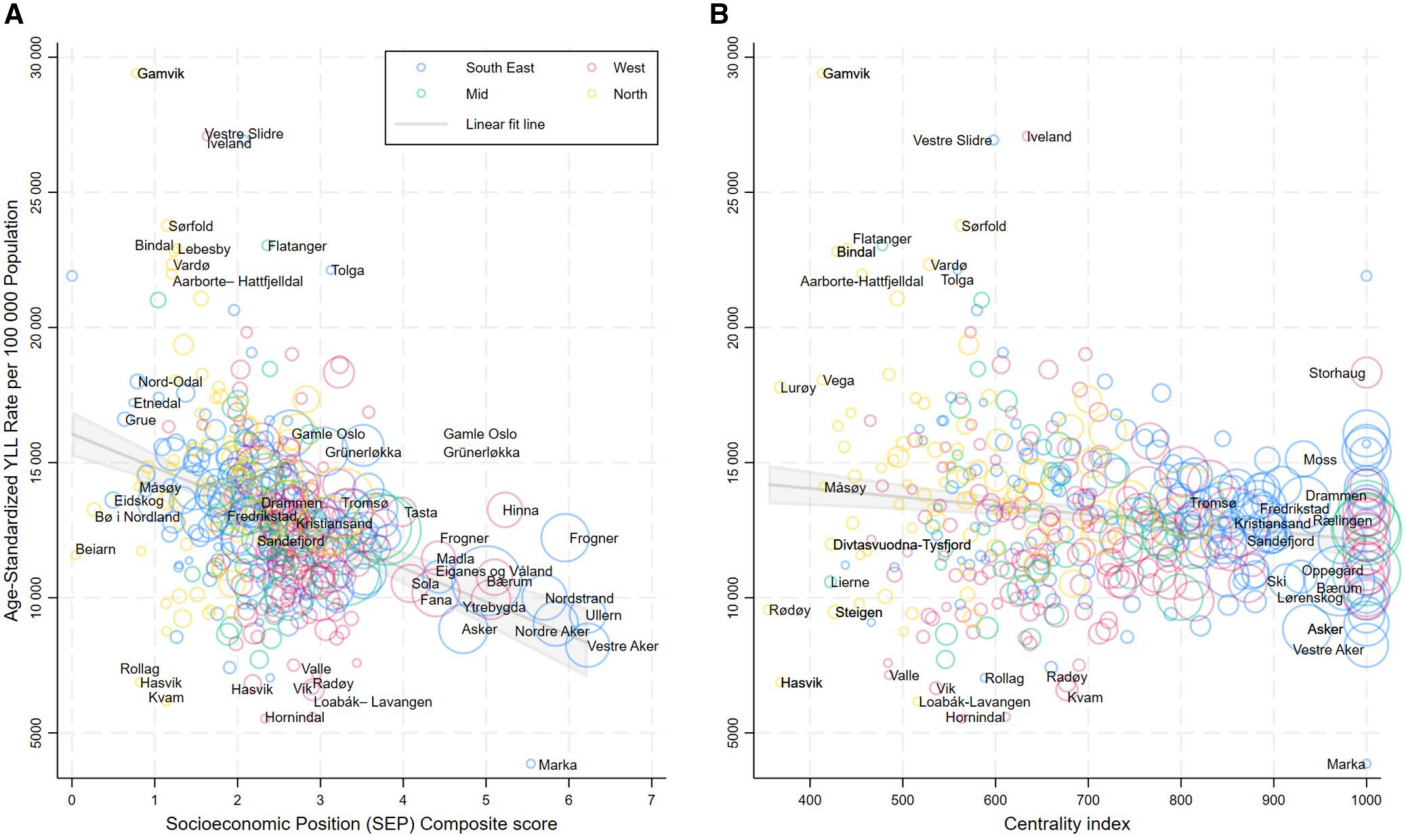
Bivariate linear associations and bubble plot of the composite SEP score (Panel A) and centrality (Panel B) each with age-standardized YLL rate per 100 000 population at the municipal and district level. Notes: The Y-axis shows the age-standardized YLL rate per 100 000 population. The size of the bubbles indicates the population size of each municipality or district. The composite SEP score, ranging from 0 to 7, combines educational attainment and income to represent local SEP, with higher scores indicating higher SEP. The centrality index is presented on a continuous scale from 0 to 1000, indicating the least to most central areas, and is used to assess the urban–rural typology.

Linear associations between the composite SEP score and age-standardized YLL rates showed a decline in YLLs with increasing SEP (Fig. 2, panel A) and centrality (panel B).

The lowest SEP quartile exhibited a 15% higher YLL rate compared to the highest SEP quartile, according to the adjusted model (IRR 1.15; 95% CI: 1.07–1.24) (Table 2). A dose–response relationship was evident, with progressively higher YLL rates observed as SEP decreased. This corresponded to an absolute difference of 2217 YLLs per 100 000 population between high and low SEP municipalities, adjusted for sex and age categories. Rural municipalities exhibited a 15% higher YLL rate (IRR 1.15; 95% CI: 1.09–1.21) compared to urban ones, after adjusting for sex and age categories. A dose–response trend was evident across all centrality categories, resulting in an absolute difference of 2057 YLLs per 100 000 population between urban and rural areas, adjusted for sex and age categories. For absolute YLL values, please refer to Supplementary Table S4.

**Table 2.** Relative and absolute associations of crude YLL rate per 100 000 population by SEP and centrality at the municipal and district level

Factor	Relative difference IRR (95% CI)		Absolute difference YLL rate per 100 000	
–	Model 1 (crude)	Model 2 (adj. age and sex)	Model 1 (crude)	Model 2 (adj. age and sex)
SEP	–	–	–	–
Q1 (high SEP, ref.)	1.00	1.00	0	0
Q2	1.21 (1.11–1.31)	1.07 (1.04–1.10)	2515 (1381–3648)	977 (622–1333)
Q3	1.32 (1.23–1.42)	1.12 (1.09–1.15)	3908 (2892–4924)	1717 (1353–2081)
Q4 (low SEP)	1.48 (1.41–1.55)	1.15 (1.07–1.24)	5859 (4798–6919)	2,127 (1000–3254)
Centrality	–	–	–	–
1 (urban, ref.)	1.00	1.00	0	0
Gr. 2 (intermediate)	1.28 (1.13–1.45)	1.08 (1.05–1.12)	3102 (1458–4746)	1154 (754–1555)
Gr. 3 (rural)	1.55 (1.35–1.78)	1.15 (1.09–1.21)	6215 (4077–8354)	2057 (1289–2825)

Notes: Municipalities and districts follow the 2019 classification, with districts being city areas in the four largest cities. SEP is evaluated via a composite score of educational attainment and income, where high SEP quartiles feature the highest levels, and low SEP quartiles the lowest. Educational attainment is the proportion with lower secondary education or less (ISCED 0–2) per municipality or district, while income is the median equivalized household income after tax. Centrality categories are derived from Statistics Norway’s 2020 classifications. Negative binomial regression models: Model 1, crude model. Model 2, adjusted for age categories and sex at the level of the municipality and district.

The PAF analysis demonstrated that 7.60% (95% CI: 7.24%–7.96%) of the YLL burden among populations in the three lower SEP quartiles could be attributed to socio-economic inequalities, when compared to the highest SEP quartile.

Sensitivity analyses of municipalities with populations over 2000, 4000, and 10 000 indicated that the individual relationships between YLLs and both SEP and centrality remained consistent across these population sizes. However, absolute differences in YLL rates diminished at higher population thresholds, indicating a convergence and resulting in more conservative estimates. For further details, refer to Supplementary Material, page 2, and Tables S3 and S4.

## Discussion

This study revealed significant inequalities in YLLs and clear associations with SEP and centrality across Norwegian municipalities. Municipalities with low SEP had a 15% higher YLL rate compared to those with high SEP. Additionally, rural municipalities also exhibited a 15% higher YLL rate compared to urban ones. The PAF analysis indicated that socio-economic inequalities accounted for 7.60% of the YLL burden in populations within SEP quartiles below the highest quartile. This relatively low PAF may reflect Norway's egalitarian society, which produces smaller health inequalities. Additionally, measuring SEP in large groups with greater variability and less precision than individual-level studies may result in smaller PAFs.

Our study confirms significant YLL inequalities among Norwegian municipalities, aligning with the 2019 GBD study by Clarsen *et al.* [3], which noted differences in crude YLLs across counties, with ‘Troms og Finnmark,’ the northernmost county, being the only one with significantly higher age-standardized YLL rates than the national average. Consistent with this, we observe that municipalities in the North health region are disproportionately represented in the low SEP quartile and exhibit the highest YLL rates. This underscores persistent regional socio-economic health disparities, especially challenging the northernmost areas. Kravdal *et al.* [16] found aggregate sociodemographic factors more strongly influenced mortality among Norwegian adults than individual factors. Similarly, our study indicates that municipal-level socio-economic disparities significantly impact mortality rates, and analysing YLLs captured both the frequency and prematurity of mortality, offering insights for targeted public health interventions beyond traditional mortality analyses. Chen-Xu *et al.* [24] studied subnational geographical and socio-economic inequalities in all-cause YLLs across the European Economic Area (2009–19), finding persistent inequalities despite a general decline. Their findings, like ours, show strong links between socio-economic factors and YLLs. While our study does not examine changes over time, the findings of higher YLLs in lower SEP quartiles align with Newton *et al.* [25], noting persisting socio-economic disparities in England from 1990 to 2013 despite reductions in YLLs and DALYs. Future research should explore temporal changes in health inequalities in Norway and compare these differences to other countries for a more comprehensive understanding. Furthermore, our findings of higher YLLs in less central municipalities align with Bremberg’s 2020 study [6], which found higher mortality in less densely populated Nordic rural areas, hypothesized to result from the limited availability of high-income job opportunities and constrained access to tertiary education in these regions.

Our study highlights the complex relationship between SEP, centrality, and premature mortality, emphasizing the interplay of spatial and socio-economic factors in health outcomes. Despite Norway’s standing as a high-income country with low-income inequality [26] and a well-educated population [27], notable variations in premature mortality related to municipal-level SEP persist. These disparities can be partly attributed to the distinct roles of income and education in shaping health outcomes. Income indirectly promotes health by providing access to resources such as preventive healthcare, nutritious food, and safe housing. Moreover, higher incomes are often associated with job stability and reduced financial stress, further supporting healthier lifestyles and mitigating health-detrimental effects [9, 28]. One proposed mechanism by which education may improve health is through enhancing health literacy and facilitating access to information, thereby increasing self-efficacy and promoting healthier habits and effective use of health resources [29]. However, attributing health outcomes solely to individual behaviours, such as educational attainment, without considering broader social determinants, may oversimplify the issue. Factors like income, employment, and living conditions, as well as marital status and social support also play crucial roles and moderates the impact of education on health outcomes [30, 31]. In this context is important to note that higher education is associated with better occupational and income prospects, which shape the socio-economic environment and enhance access to essential health resources [9]. The interconnectedness of these factors results in complex socio-economic health dynamics, where poor health can negatively impact income and education, highlighting their reciprocal relationship [9]. Individual-level variations intersect with broader environmental and community conditions, impacting residents in diverse ways. Living in disadvantaged areas marked by limited resources, inadequate infrastructure, and scarce economic opportunities can disproportionately impact the health of poorer individuals, who often rely more heavily on shared community resources [32]. Moreover, environmental challenges such as pollution, noise, and substandard housing exacerbate existing health risks and elevate mortality rates, underscoring the compounded effects of socio-economic and environmental determinants on health [32].

Although our findings highlight substantial socio-economic disparities in YLL across municipalities, there is likely greater heterogeneity within individual city districts that our analysis does not fully capture, as it primarily focuses on broad SEP categories like income and education. Exploring more granular levels of resource deprivation could reveal different patterns of socio-economic disparities, particularly in larger cities like Oslo and Bergen, where these inequalities are pronounced [5].

Our study revealed significant geographical disparities in premature mortality, with higher YLLs in rural areas. Age emerged as an important confounder, likely reflecting societal trends where younger, educated individuals often migrate to urban regions, leaving rural areas with older, less educated populations at greater risk. However, other factors—such as socio-economic conditions and varying economic opportunities across regions—likely contribute, as suggested by consistent results even after adjusting for age. Urban areas like Oslo, with diverse economic activities, and western regions like Stavanger and Bergen, fuelled by the oil sector, generally have higher incomes compared to rural regions [33]. These urban regions are characterized by high SEP in our study, contrasting with rural areas facing socio-economic challenges. Despite generally lower living costs, rural areas often struggle with poverty, limited healthcare, and social isolation adversely impacting mortality [5]. This is exacerbated by occupational hazards in industries like agriculture and fishing [34]. Lower educational levels may also limit occupational mobility, confining individuals to high-risk jobs. Additionally, rural populations are at increased risk of fatal injuries due to greater injury severity, delayed emergency response times, and limited access to specialized services [35]. Regional differences in health behaviours, including diet, physical activity, and smoking, also significantly impact mortality rates [4]. Smoking is particularly prevalent among lower socio-economic groups and older generations in less central areas, continuing as a major factor in mortality in Norway with long-term effects often appearing decades after exposure [36].

### Methodological considerations

This study has several strengths. Firstly, it utilizes high-quality data from national registries, covering the entire Norwegian population. By applying consistent criteria and methodologies for municipal and district-level analyses and including only populations over 1000, the study enhances representativeness and generalizability within the Norwegian context while ensuring comparability across spatial scales. Additionally, incorporating district-level data for the four largest cities, which account for 25% of Norway’s population, provides more granular insights. The study captures various aspects of SEP by using broad indicators such as educational attainment and income, and integrating these factors into a composite score enhances the analytical depth, supported by demonstrated internal consistency and reliability [19]. Assessing both relative and absolute inequalities offers a comprehensive view of disparities [37].

The are limitations that need to be addressed. The study’s ecological design prevents distinguishing between individual and group-level effects, necessitating caution to avoid ecological fallacies [38]. Although unmeasured factors such as occupation, employment, and wealth correlate with the SEP factors included in this study, they might still account for some variations in mortality. It is also important to acknowledge that we measured income, not wealth inequality. Pensioners, who often have low incomes but may have accumulated wealth, might be unequally represented in the lower quartile, which may not accurately reflect their SEP. However, adjusting for age as a covariate accounts for most pensioners in the 70+ age groups. Assessing health outcomes using socio-economic variables can be challenging, as these variables may change in response to health conditions, posing a risk of reverse causation [38, 39]. Given the observational nature of this study, it is essential to interpret the results as associational rather than causal. While the PAF measure should be interpreted cautiously, as it assumes a causal relationship that cannot be verified in an observational study, it indicates substantial impact of socio-economic inequalities on premature mortality [38]. The cross-sectional design uses 2019 data, limiting temporal insights. This baseline for Norway may not be applicable to countries with different welfare and healthcare systems. Additionally, YLL estimates are not directly comparable to other studies using GBD methodologies, as the Norwegian population is the standard reference.

To conclude, significant inequalities in premature mortality are evident across Norwegian municipalities, closely linked to population-level SEP and centrality. This study illustrates the feasibility of using burden of disease methods to assess these inequalities at the local level and underscores the importance of multidimensional public health strategies to address the intricate interplay of socio-economic and demographic factors impacting health outcomes. To improve overall health and reduce premature mortality, policymakers should enhance municipal health services by tailoring them to meet the specific needs of different communities, ensuring that interventions are aligned with their unique health determinants and risk factors. Balancing specialized, individual-focused measures with broad, integrative approaches is crucial for effectively addressing health disparities, ensuring that everyone benefits from health services while those with greater needs receive more support [40]. Future research should emphasize cause-specific outcomes to precisely identify the relative burden of different causes of premature deaths. Utilizing longitudinal data can reveal patterns and more effectively guide resource allocation and preventive measures, enabling more effective, targeted interventions to reduce health inequalities at the local level in Norway.

## Supplementary Material

ckaf086_Supplementary_Data

## Data Availability

The data used in this study are not publicly available, as they are sourced from Statistics Norway. Access to these data may be obtained through Statistics Norway by following their data access procedures. Key points Significant relative and absolute inequalities in premature mortality persist across Norwegian municipalities and districts, despite Norway’s protective societal structures. There are significant associations between low SEP and rural typology with premature mortality at these local levels. Our study demonstrates the feasibility of using burden of disease methods to assess geographical and socio-economic inequalities in premature mortality at the local level. Policymakers should develop targeted public health interventions aimed at municipalities and districts with higher levels of premature mortality, particularly those with low SEP and rural typologies.
