# Supplementary material for: Geographical and socio-economic inequalities in years of life lost across Norwegian municipalities and city districts in 2019: an ecological registry-based study
Source: Eur J Public Health. 2025 Sep 18;35(6):1100–6. doi: 10.1093/eurpub/ckaf086 (PMC12707486; doi:10.1093/eurpub/ckaf086)
Supplement: ckaf086_Supplementary_Data [file ckaf086_supplementary_data.docx]

**Supplementary material**

Methods

**Socioeconomic variables**

Educational attainment and income were evaluated individually as well as integrated into a composite score. To capture the combined effect of these key socioeconomic variables, we constructed a composite SEP score that combined educational attainment and income, following guidelines from the Handbook on Constructing Composite Indicators^1^. The process included: 1) aligning measure directions so higher values indicated higher SEP (e.g. higher levels of education and income); 2) assessing normality and identifying and trimming outliers; 3) standardizing low education and income using z-scores (mean=0, SD=1) for comparability; 4) aggregating the indicators using the arithmetic mean; and 5) adding a constant (equal to the minimum value) to eliminate negative values. Both indicators were equally weighted. The Pearson correlation was 0.55, item-total correlations were 0.47 for each indicator, and Cronbach’s alpha coefficient was 0.72, demonstrating good internal reliability.

**Statistical Analysis**

**Model assessment**

We evaluated both Poisson and negative binomial regression models. Due to overdispersion in the outcome variable, Years of Life Lost (YLLs)—where variance exceeded the mean—we opted for the negative binomial model. Goodness-of-fit tests supported this choice: the Poisson model exhibited a large chi-square value and significant p-value in the Hosmer-Lemeshow test, indicating poor fit, and higher Akaike Information Criterion (AIC) and Bayesian Information Criterion (BIC) values compared to the negative binomial model. Additionally, the log pseudolikelihood was lower for the Poisson model, further justifying our selection of the negative binomial approach.

The specification for the mixed-effects negative binominal regression analysis adjusted for age structure and sex was conducted using the Stata command, with the following specification:

YLLs*ij*​ = *β*0 ​+ *β*1SEP*ij*​ + *β*2​AgeGroup*ij*​ + *β*3​ProportionFemales*ij*​ + *uj* ​+ *ϵij*​

Where:

- YLLs*ij*​ is the outcome variable (Years of Life Lost) for municipality *i* in health region *j*.
- *β*0​ is the intercept.
- *β*1​ is the coefficient for the composite score for socioeconomic position (SEP).
- *β*2​ is the coefficient for the 10-year age group composition.
- *β*3​ is the coefficient for the proportion of females.
- *uj*​ is the random intercept for health region j*j*.
- *ϵij*​ is the error term.

**Sensitivity analysis**

To evaluate the robustness of the composite SEP score, we conducted a sensitivity analysis, exploring various weighting structures between education and income.

To ensure robustness and assess potential bias introduced by small population sizes, we conducted sensitivity analyses by excluding municipalities and districts with fewer than 2000, 4000, and 10 000 residents for SEP and fewer than 2000 and 4000 for centrality, since rural and intermediate populations did not exceed 10 000. Smaller populations are often subject to greater statistical variability, which can lead to disproportionate influence from outliers or irregular patterns, potentially skewing the results. By implementing these population thresholds, the analyses aimed to mitigate such biases, enhance the reliability of the findings, and improve the generalizability of the conclusions across larger, more stable populations.

**Population Attributable Fraction (PAF)**

The PAF was derived using the following formula^2^:

$$PAF=\frac{\Sigma_{i}P_{i}\left( RR_{i}-1 \right)}{\Sigma_{i}P_{i}\left( RR_{i}-1 \right)+1}$$

where P_i_​ represents the proportion of the population in each SEP category, and RR_i_​ denotes the relative risk of YLLs for each category compared to the high SEP quartile (reference). The exposure distribution was based on population proportions across SEP quartiles, with relative risks from age- and sex-adjusted IRRs from mixed-effects negative binomial regression models. The analysis assumes that if socioeconomic disparities were equalised to the reference SEP level, the YLL burden could be reduced by a proportion equivalent to the PAF. The 95% confidence intervals for the PAF were calculated using the Approximate Delta Method, considering the variance of log(IRRs) and exposure proportions.

Results

**Main analysis**

Supplementary table 1 presents the relative associations of crude Years of Life Lost (YLL) rates per 100 000 population by educational attainment and income at the municipal and district level. For comparison, associations of crude YLL rates by SEP are also included. In Model 2, which adjusts for age categories and sex, lower educational attainment, lower income, and lower SEP are all associated with higher YLL rates, with the strongest associations observed with income and SEP.

**Supplementary table 1.** Relative associations of crude Years of Life Lost (YLL) rate per 100 000 population by education, income, and SEP at the municipal and district level

| **Factor** | **Relative difference**  **IRR (95% CI)** | |
| --- | --- | --- |
|  | **Model 1**  **(crude)** | **Model 2**  **(adj. age & sex)** |
| **Education** |  |  |
| Q1 (high, ref.) | 1.00 | 1.00 |
| Q2 | 1.14 (1.06-1.22) | 1.06 (1.00-1.12) |
| Q3 | 1.20 (1.12-1.29) | 1.07 (1.01-1.14) |
| Q4 (low) | 1.42 (1.32-1.52) | 1.09 (1.02-1.18) |
| **Income** |  |  |
| Q1 (high, ref.) | 1.00 | 1.00 |
| Q2 | 1.16 (1.09-1.25) | 1.06 (1.00-1.11) |
| Q3 | 1.26 (1.17-1.36) | 1.10 (1.04-1.17) |
| Q4 (low) | 1.42 (1.31-1.53) | 1.15 (1.07-1.24) |
| **SEP** |  |  |
| Q1 (high SEP, ref.) | 1.00 | 1.00 |
| Q2 | 1.21 (1.11–1.31) | 1.07 (1.04–1.10) |
| Q3 | 1.32 (1.23–1.42) | 1.12 (1.09–1.15) |
| Q4 (low SEP) | 1.48 (1.41–1.55) | 1.15 (1.07–1.24) |

**Notes**: Municipalities and districts follow the 2019 classification, with districts being city areas in the four largest cities. Educational attainment is the proportion with lower secondary education or less (ISCED 0-2) per municipality or district, while income is the median equivalised household income after tax. SEP is evaluated via a composite score of educational attainment and income, where high SEP quartiles feature the highest levels, and low SEP quartiles the lowest. Negative binomial regression models: Model 1: crude model. Model 2: adjusted for age categories and sex at the level of the municipality and district.

**Sensitivity analysis**

The sensitivity analysis of different weights for education and income for the adjusted model revealed that the differences in IRR values across various weight combinations are generally small (Supplementary figure 1 and table 2. Specifically, the comparison between quartiles (Q2, Q3, Q4) shows minimal variation, with IRR values for Q4 ranging from 1.15 to 1.17 across all weight scenarios. Additionally, the large overlap of confidence intervals between different weights suggests that the choice of weights does not lead to significant changes in the results or interpretation, indicating that using equal weights provided similar outcomes to other weight combinations of the composite score.

**Supplementary figure 1.** Incidence rate ratios for each quartile for different weights of education and income


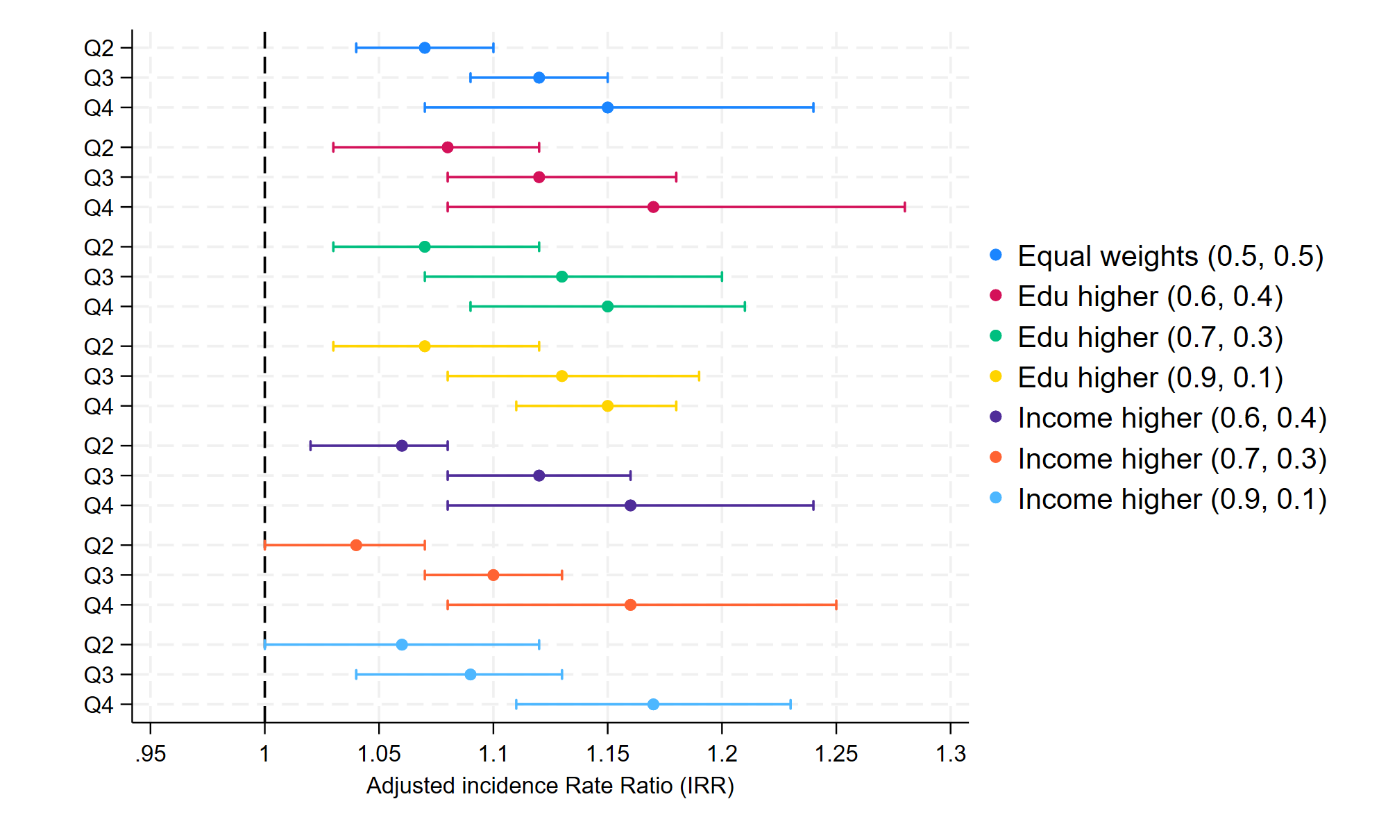
Note: all estimates based on the final adjusted model using a random intercept by health region. The reference group is quartile 1 (the lowest socioeconomic group).

**Supplementary table 2.** Incidence rate ratios and absolute differences for each quartile for different weights of education and income

| **Weight Group** | **Quartile** | **IRR (95% CI)** | **Difference (95% CI)** |
| --- | --- | --- | --- |
| Equal weights (0.5, 0.5) | Q2 | 1.07 (1.04, 1.10) | 977 (622, 1333) |
|  | Q3 | 1.12 (1.09, 1.15) | 1717 (1353, 2081) |
|  | Q4 | 1.15 (1.07, 1.24) | 2127 (1000, 3254) |
| Edu higher (0.6, 0.4) | Q2 | 1.08 (1.03, 1.12) | 1065 (475, 1656) |
|  | Q3 | 1.12 (1.08, 1.18) | 1697 (1123, 2272) |
|  | Q4 | 1.17 (1.08, 1.28) | 2385 (1093, 3677) |
| Edu higher (0.7, 0.3) | Q2 | 1.07 (1.03, 1.12) | 1023 (459, 1586) |
|  | Q3 | 1.13 (1.07, 1.20) | 1853 (1067, 2639) |
|  | Q4 | 1.15 (1.09, 1.21) | 2013 (1238, 2789) |
| Edu higher (0.9, 0.1) | Q2 | 1.07 (1.03, 1.12) | 1018 (432, 1603) |
|  | Q3 | 1.13 (1.08, 1.19) | 1841 (1148, 2534) |
|  | Q4 | 1.15 (1.11, 1.18) | 2015 (1634, 2395) |
| Income higher (0.6, 0.4) | Q2 | 1.06 (1.02, 1.08) | 657 (284, 1030) |
|  | Q3 | 1.12 (1.08, 1.16) | 1650 (1106, 2194) |
|  | Q4 | 1.16 (1.08, 1.24) | 2192 (1086, 3297) |
| Income higher (0.7, 0.3) | Q2 | 1.04 (1.00, 1.07) | 532 (77, 987) |
|  | Q3 | 1.10 (1.07, 1.13) | 1428 (1108, 1729) |
|  | Q4 | 1.16 (1.08, 1.25) | 2301 (1170, 3432) |
| Income higher (0.9, 0.1) | Q2 | 1.06 (1.00, 1.12) | 859 (60, 1659) |
|  | Q3 | 1.09 (1.04, 1.13) | 1199 (587, 1811) |
|  | Q4 | 1.17 (1.11, 1.23) | 2402 (1707, 3097) |

Note: all estimates based on the final adjusted model using a random intercept by health region. The reference group is quartile 1 (the lowest socioeconomic group).

Supplementary table 3 presents the relative associations of Years of Life Lost (YLL) rates per 100 000 population by socioeconomic position (SEP) and centrality at the municipal and district level. The patterns observed in the main analysis remained consistent across different population thresholds.

For SEP, the incidence rate ratios (IRRs) indicated a steady increase in YLL rates as SEP decreased, with similar IRRs noted regardless of the minimum population size used in the analysis. This consistency demonstrated that the association between lower SEP and higher premature mortality rates was not significantly influenced by the size of the population examined, affirming the robustness of our main findings.

Similarly, for centrality, the higher IRRs in more rural areas remained evident across the various population thresholds. Importantly, for centrality, sensitivity analyses for populations exceeding 10 000 are not applicable, as both rural and intermediate groups fall below this threshold. Although some slight variations in the IRR values occurred, the overall trend of increased mortality risk in less central areas persisted. This consistency across different population filters reinforces the reliability of the associations observed in the main analysis, underscoring their generalizability to larger and more stable populations, thereby strengthening the validity of our conclusions regarding the impact of SEP and centrality on premature mortality rates.

**Supplementary table 3.** Relative associations of Years of Life Lost (YLL) rate per 100 000 population by SEP and centrality at the municipal and district level

| **Factor** | **Main analysis**  **Pop. > 1000,**  **adj. age & sex** | **Sensitivity**  **Pop. > 2000,**  **adj. age & sex** | **Sensitivity**  **Pop. > 4000,**  **adj. age & sex** | **Sensitivity**  **Pop. > 10 000,**  **adj. age & sex** |
| --- | --- | --- | --- | --- |
|  | **IRR (95% CI)** | **IRR (95% CI)** | **IRR (95% CI)** | **IRR (95%CI)** |
| **SEP** |  |  |  |  |
| Q1 (high SEP, ref.) | 1.00 | 1.00 | 1.00 | 1.00 |
| Q2 | 1.07 (1.04–1.10) | 1.07 (1.04–1.10) | 1.06 (1.05–1.07) | 1.07 (1.05–1.09) |
| Q3 | 1.12 (1.09–1.15) | 1.12 (1.09–1.15) | 1.12 (1.06–1.19) | 1.13 (1.07–1.20) |
| Q4 (low SEP) | 1.15 (1.07–1.24) | 1.15 (1.07–1.24) | 1.14 (1.02–1.28) | 1.15 (1.09–1.22) |
| **Centrality** |  |  |  |  |
| Group 1(urban, ref.) | 1.00 | 1.00 | 1.00 |  |
| Group 2 (intermediate) | 1.08 (1.05–1.12) | 1.08 (1.02–1.14) | 1.08 (1.03–1.12) |  |
| Groups 3 (rural) | 1.15 (1.09–1.21) | 1.13 (1.05–1.20) | 1.12 (1.05–1.19) |  |

**Notes**: YLL per 100 000 population represents the mean crude rate. Municipalities and districts follow the 2019 classification, with districts being city areas in the four largest cities. SEP is evaluated via a composite score of educational attainment and income, where high SEP quartiles feature the highest levels, and low SEP quartiles the lowest. Educational attainment is the proportion with lower secondary education or less (ISCED 0-2) per municipality or district, while income is the median equivalised household income after tax. Centrality categories are derived from Statistics Norway's 2020 classifications**.** Negative binomial regression model: Model 2 (main): adjusted for age categories and sex**.** For centrality, sensitivity analyses for populations exceeding 10 000 are not applicable, as both rural and intermediate groups fall below this threshold.

Supplementary table 4 displays the absolute Years of Life Lost (YLL) rates per 100 000 population and the absolute differences, by socioeconomic position (SEP) and centrality at the municipal and district level. In terms of absolute values and differences, the highest SEP quartile (Q1) consistently exhibited the lowest YLL rates, starting at 13 868 per 100 000 for populations over 1000 and decreasing to 12 126 for those over 10 000 (Table S4). In contrast, YLL rates were progressively higher across the other quartiles, with the lowest SEP quartile (Q4) having the highest rates, beginning at 15 995 and dropping to 13 951 per 100 000 as the population size increased from 1000 to 10 000. The absolute differences in YLL rates between quartiles showed that Q2, Q3, and particularly Q4 had significantly higher YLLs compared to Q1. Although these differences were most pronounced in smaller population groups, they tended to diminish somewhat as the population threshold increased. This pattern suggests that while higher SEP is associated with reduced YLLs, the disparities lessen slightly in larger population samples. Urban areas (Group 1) consistently experienced the lowest YLL rates, starting at 13 689 per 100 000 in the main analysis and declining to 12 299 at the largest population threshold (Table S3). Intermediate (Group 2) and rural areas (Group 3) exhibited higher YLL rates, with rural areas initially showing the highest rates. These rates decreased from 15 746 for populations over 1000 to 12 719 for populations over 10 000.

The disparities in YLL rates between rural and urban areas, indicated by the absolute differences, were consistent with the main analysis results. However, these differences diminished as the population threshold increased, suggesting a convergence in YLL rates when examining larger population groups.

**Supplementary table 4.** Absolute Years of Life Lost (YLL) rate per 100 000 population and absolute differences, by SEP and centrality at the municipal and district level

| **Factor** | **Main, pop. > 1000,**  **adjusted age & sex**  **YLL rates per**  **100 000 (95% CI)** | **Sens, pop. > 2000,**  **adjusted age & sex**  **YLL rates per**  **100 000 (95% CI)** | **Sens, pop. > 4000,**  **adjusted age & sex**  **YLL rates per**  **100 000 (95% CI)** | **Sens, pop. > 10 000,**  **adjusted age & sex**  **YLLs rates per**  **100 000 (95% CI)** |
| --- | --- | --- | --- | --- |
| **SEP** |  |  |  |  |
| *Absolute value* |  |  |  |  |
| Q1 (high SEP, ref.) | 13 868 (13 295-14 442) | 13 456 (12 914-13 999) | 12 865 (11 895-13 834) | 12 126 (11 174-13 078) |
| Q2 | 14 846 (14 355-15 336) | 14 341 (13 888-14 794) | 13 611 (12 612-14 610) | 12 968 (11 794-14 361) |
| Q3 | 15 585 (15 249- 15 921) | 15 047 (14 951-15 144) | 14 454 (13 946-14 610) | 13 748 (13 260-14 668) |
| Q4 (low SEP) | 15 995 (15 272-16 719) | 15 117 (14 804-15 431) | 14 707 (13 914-15 501) | 13 951 (13 585-14 361) |
| *Absolute difference* |  |  |  |  |
| Q1 (high SEP, ref.) | 0 | 0 | 0 | 0 |
| Q2 | 977 (622-1333) | 885 (555-1214) | 746 (591-901) | 842 (560-1125 |
| Q3 | 1717 (1353-2081) | 1591 (1014-2168) | 1590 (818-2361) | 1622 (902-2342) |
| Q4 (low SEP) | 2127 (1000-3254) | 1661 (838-2484) | 1843 (275-3410) | 1824 (1148-2501) |
| **Centrality** |  |  |  |  |
| *Absolute value* |  |  |  |  |
| Group 1(urban, ref.) | 13 689 (13 222-14 156) | 13 358 (12 847-13 870) | 12 795 (12 536-13 054) |  |
| Group 2(intermed.) | 14 844 (14 577-15 111) | 14 425 (14 092-14 757) | 13 765 (13 436-14 095) |  |
| Group 3 (rural) | 15 746 (15 202-16 290) | 15 003 (14 505-15 561) | 14 291 (13 567-15 007) |  |
| *Absolute difference* |  |  |  |  |
| Group 1(urban, ref.) | 0 | 0 | 0 |  |
| Group 2(intermed.) | 1154 (754-1555) | 1066 (286-1847) | 970 (387-1554) |  |
| Group 3 (rural) | 2057 (1289-2825) | 1674 (710-2639) | 1496 (583-2410) |  |

**Notes**: YLL per 100 000 population represents the mean crude rate. Municipalities and districts follow the 2019 classification, with districts being city areas in the four largest cities. SEP is evaluated via a composite score of educational attainment and income, where high SEP quartiles feature the highest levels, and low SEP quartiles the lowest. Educational attainment is the proportion with lower secondary education or less (ISCED 0-2) per municipality or district, while income is the median equivalised household income after tax. Centrality categories are derived from Statistics Norway's 2020 classifications**.** Negative binomial regression model: Model 2 (main): adjusted for age categories and sex**.** For centrality, sensitivity analyses for populations exceeding 10 000 are not applicable, as both rural and intermediate groups fall below this threshold.

## References

1 Nardo M, Saisana M, SaltelliI A, Tarantola S, Hoffmann A, E G. Handbook on Constructing Composite Indicators: Methodology and User Guide. <https://knowledge4policy.ec.europa.eu/publication/handbook-constructing-composite-indicators-methodology-user-guide-0_en#:~:text=This%20Handbook%20aims%20to%20provide%20a%20guide%20to%20the%20construction> Accessed 19 December 2024.

2 HealthStats NSW. Population attributable fractions (PAFs). <https://www.healthstats.nsw.gov.au/Resources/pafs.pdf> Accessed 23 January 2025.
